# The Compulsive Exercise Test: confirmatory factor analysis and links with eating psychopathology among women with clinical eating disorders

**DOI:** 10.1186/s40337-016-0113-3

**Published:** 2016-08-19

**Authors:** Caroline Meyer, Carolyn R. Plateau, Lorin Taranis, Nicola Brewin, Jackie Wales, Jon Arcelus

**Affiliations:** 1 WMG, University of Warwick, Coventry, CV4 7AL UK; 2 Warwick Medical School, Coventry, CV77AL UK; 3 University Hospitals Coventry and Warwickshire NHS Trust, Coventry, UK; 4 School of Sport, Exercise and Health Sciences, Loughborough University, Loughborough, UK; 5 Leicestershire Adult Eating Disorders Service, Leicestershire Partnership NHS Trust, Bennion Centre, Leicester, UK; 6 Nottingham Centre for Gender Dysphoria, Nottingham, UK; 7 Division of Psychiatry and Applied Psychology, Faculty of Medicine & Health Sciences, University of Nottingham, Nottingham, UK

**Keywords:** Compulsive exercise, Eating disorders, Measurement, Detection, Screening

## Abstract

**Background:**

This study aimed to determine the psychometric properties of the Compulsive Exercise Test (CET) among an adult sample of patients with eating disorders.

**Method:**

Three hundred and fifty six patients and 360 non-clinical control women completed the CET and the Eating Disorders Examination questionnaire (EDE-Q).

**Results:**

A confirmatory factor analysis revealed that the clinical data showed a moderate fit to the previously published five factor model derived from a community sample (Taranis L, Touyz S, Meyer C, Eur Eat Disord Rev 19:256-268, 2011). The clinical group scored significantly higher than the non-clinical group on four of the five CET subscales, and logistic regression analysis revealed that the CET could successfully discriminate between the two groups. A Receiver Operating Curve analysis revealed that a cut-off score of 15 on the CET resulted in acceptable values of both sensitivity and specificity.

**Conclusions:**

The CET appears to have a factor structure that is acceptable for use with an adult sample of patients with eating disorders. It can identify compulsive exercise among patients with eating disorders and a cut-off score of 15 is acceptable as indicating an appropriate cut-off point.

## Background

Compulsive exercise is an important component of eating psychopathology. Previously, the Compulsive Exercise Test (CET) was developed to assess this characteristic. This study aimed to determine the psychometric properties of the Compulsive Exercise Test (CET) among an adult sample of patients with eating disorders.

A recent review reported that up to 85 % of eating disordered patients engage in compulsive exercise [16]. Compulsive exercise has been defined as a rigid and highly driven urge to be physically active, in association with a perceived inability to stop exercising despite the individual being aware of the possible negative consequences [45]. Patients who engage in such behaviour tend to have a worse outcome and require longer hospitalisation than non-exercising patients (e.g., [6, 11, 41]). Therefore, it is essential that we can adequately operationalise and measure this construct within the context of eating disorder patients.

Until relatively recently, compulsive exercise had been conceptualised as a uni-dimensional construct (typically centred on its utility in managing shape and weight; e.g., [13]). In addition, it had often been measured via quantitative (i.e. frequency, duration and intensity of exercise), rather than qualitative metrics (i.e. the psychological experience of exercise; [32]). However, it is clear from numerous research studies that this early conceptualisation is inadequate to fully assess the underlying features of compulsive exercise within the context of the eating disorders [33]. Specifically, in addition to the control of weight and shape, compulsive exercise involves components of both mood improvement (positive reinforcement) and affective avoidance (negative reinforcement; e.g., [8, 9]). Exercising is commonly associated with positive affect, and models of exercise dependence have suggested that exercise-induced euphoric states are highly positively reinforcing [19]. However, exercising for negative affect regulation has been consistently identified as contributory factor to the development and maintenance of eating disorders [22, 46]. Specifically, experiencing affective withdrawal symptoms when unable to exercise has been identified as a central characteristic of psychological dependence on exercise [21]. The experience of withdrawal symptoms from exercise has been shown to discriminate between eating disordered and non-clinical groups [7]. In addition, compulsive exercise is typified by a compulsive rigidity and a lack of intrinsic enjoyment (e.g., [4, 7, 34]).

In recognition of the multi-dimensional nature of compulsive exercise, a new self-report measure; The Compulsive Exercise Test (CET) was developed and initially psychometrically tested within a group of non-clinical exercising women [45]. The CET is based on a maintenance model that was developed in the context of the eating disorders [33], and measures compulsive exercise in a multi-dimensional way. An initial factor analysis conducted with the CET identified five distinct subscales [45]: *Avoidance and rule-driven behaviour* (continuing to exercise despite injury or illness, making up for missed sessions, experiencing exercise withdrawal symptoms, and feeling extremely guilty when unable to exercise); *Weight control exercise* (exercising to modify or control weight and shape, engaging in compensatory exercise to account for calorie intake); *Mood improvement* (experiencing the positive, mood enhancing effects of exercise); *Lack of exercise enjoyment* (experiencing exercise as a chore rather than a pleasure), and *Exercise rigidity* (maintaining a strict and repetitive exercise schedule). Subsequently, this initial factor structure was supported for use with a community sample of male and female adolescents [20], and a slightly revised version has been published for use with athletes [39]. A preliminary, relatively small sample sized (*n* = 104) study used Structural Equation Modelling to determine the robustness of the factor structure within a group of adolescents with eating disorders [18]. Using relatively stringent criteria, the study found that the original factor structure did not hold, and as yet, no alternative factor structure has been identified for use with clinical adolescents. However, using the original factor structure [45], Formby et al. found that the CET reliably distinguished the clinical and non-clinical groups and supported the multi-dimensional model of compulsive exercise in this group of adolescent patients.

In summary, compulsive exercise is an important behaviour, due to its prevalence within both clinical and non-clinical groups. The CET is based on a multi-dimensional conceptualisation of such exercise, which takes into account emotional and cognitive aspects [33]. Over 30 studies have already been published using the CET, despite its psychometric rigour remaining unestablished within an adult clinical sample. The purpose of this study was to test the psychometric properties of the CET among a group of adult patients with eating disorders.

The study had three aims. First, to determine whether the previously reported factor structure of the CET, derived and replicated in community-based studies, is appropriate for use with an adult clinical group. Second, to explore differences in CET scores between the clinical and control groups, and to assess the discriminative validity of the CET in distinguishing between clinical and control participants. It was predicted that eating disorder patients would score significantly higher on CET scores than controls. Finally, to determine an appropriate cut-off score to distinguish between clinical cases with and without compulsive exercise.

## Method

### Participants

#### Clinical group

Clinical participants were 356 female patients aged 16–60 (mean = 27.6, SD = 9.65) all of whom met criteria for an eating disorder as specified in the Diagnostic and Statistical Manual of Mental Disorders (DSM-IV; [2]). The diagnosis was provided by a Consultant Psychiatrist via a standardized interview in all cases. The diagnostic distribution was: 25.9 % anorexia nervosa (AN); 31 % bulimia nervosa (BN); 38 % eating disorder not otherwise specified (EDNOS); and 5 % binge eating disorder (BED). Of AN patients, 71 % were restrictive subtype, and of BN patients, 72 % were purging subtype. See Table 1 for full demographics. Participants were recruited from three specialist eating disorder services in the UK. Participation was voluntary and all patients completed the measures either as part of their initial assessment or were mailed a study pack via post containing: an information sheet describing the nature and purpose of the study; a consent form; the study measures; and a return envelope.

**Table 1:** Characteristics of the sample and comparison of CET and EDE-Q scores across clinical and control groups

	Group				Mann Whitney U (z)	Effect size (*r*)
	Clinical (*n* =356)		Control (*n* = 360)			
	α	Mean (SD)	α	Mean (SD)		
BMI	–	21.2 (8.07)	–	22.1 (4.23)	5.05*	0.19
Age	–	27.6 (9.65)	–	25.8 (7.49)	1.18	0.04
EDEQ						
Global	0.72	4.40 (1.16)	0.85	1.30 (1.06)	21.1*	0.79
Restraint	0.73	4.09 (1.71)	0.75	1.21 (1.22)	18.5*	0.69
Eating concern	0.73	3.93 (1.39)	0.61	0.66 (0.089)	21.1*	0.79
Shape concern	0.83	4.95 (1.38)	0.73	1.84 (1.40)	20.3*	0.76
Weight concern	0.63	4.61 (1.46)	0.85	1.48 (1.31)	20.0*	0.75
CET						
Global	0.93	14.6 (4.71)	0.87	11.4 (3.37)	9.88*	0.37
Avoidance	0.96	2.75 (1.71)	0.91	1.74 (1.28)	8.13*	0.30
Weight control	0.77	3.47 (1.34)	0.83	2.59 (1.17)	9.23*	0.34
Mood improvement	0.87	3.37 (1.28)	0.82	3.26 (1.12)	1.49	0.06
Lack of exercise enjoyment	0.62	2.21 (1.19)	0.85	1.48 (1.09)	7.67*	0.29
Exercise rigidity	0.82	2.90 (1.55)	0.76	2.37 (1.21)	5.41*	0.20

*p < 0.001

#### Control group

The control group comprised 360 women aged 16–60 years (*M* = 25.8, *SD* = 7.49). The women were recruited from a variety of different settings, including a University campus, workplaces and sports clubs. None of the participants were elite or sub-elite athletes. Control participants who reported having a current or historical eating disorder were excluded from the sample. Control participants were recruited by opportunity sampling, either via email circulation lists or directly by the researcher. Each participant completed the anonymous questionnaire alone and returned it to the researcher.

### Measures and procedure

Following ethical approval and informed consent, all participants completed the following self-report instruments, as part of a broader set of assessment measures.

#### The Eating Disorders Examination Questionnaire (EDE-Q version 6.0; [15])

The EDE-Q is a comprehensively utilised, well validated and reliable measure of eating pathology comprising of 36 self-report items. The measure focuses on the preceding 28 days, and the items assess the main behavioural and attitudinal features of eating disorders. It has four subscales: restraint, eating concern, shape concern and weight concern. Higher scores on the measure reflect increased eating pathology. These subscales have previously demonstrated good psychometric properties with Cronbach’s alpha coefficients ranging from 0.78 to 0.93 and test-retest correlations ranging from 0.87 to 0.91 [25, 36]. Frequencies of eating-disordered behaviours are assessed in terms of the number of episodes occurring in the past 4 weeks. These items do not contribute to subscale scores. The EDE-Q assesses frequency of objective episodes of overeating involving loss of control (Objective binge). In addition, the EDE-Q assesses the frequency of compensatory behaviours: self-induced vomiting, laxative abuse, and driven exercise to control weight or shape.

#### The Compulsive Exercise Test (CET; [45])

The CET is comprised of 24 self-report items that are designed to assess the core cognitive, behavioural and emotional features of compulsive exercise. Items are rated on a 6-point Likert type scale from 0 (*never true*) to 5 (*always true*) and generates five subscales: Avoidance and rule-driven behaviour, Weight control exercise, Mood improvement, Lack of exercise enjoyment, and Exercise rigidity. Higher scores on the CET is indicative of greater pathology. The CET has previously demonstrated good psychometric properties in non-clinical samples with excellent concurrent and convergent validity, and Cronbach’s alpha coefficients ranging from 0.72 to 0.88 [20, 42] Reliability coefficients for the current study are given in Table 1.

### Data analysis

The small proportion of missing data (<5 %) was within an acceptable threshold [40]. Where there were missing data, calculation of EDE-Q subscale and global scores, followed the methods recommended by Fairburn and Beglin [15] namely, for those subscales where half or more of the data were available, the subscale score was calculated by taking the mean of the available items. Global EDE-Q was only calculated when scores on more than half (i.e., 3 or 4) of the four subscales were available. The same method was used to calculate CET subscale scores where there were missing data.

In order to test the first aim, the clinical data were subjected to a Confirmatory Factor Analysis (CFA). This was deemed appropriate as a five-factor structure of the CET has been previously demonstrated as appropriate with both adolescent and adult community samples (e.g., [20, 45]), and is an approach commonly utilized within the literature (e.g., [12, 18]). First, the data were assessed for normality and screened for outliers. Two multivariate outliers were subsequently removed (d^2^ = 95.93; 79.64; *p*1 < 0.00; *p*2 < 0.00 in both cases), leaving a total sample of 354. Bootstrapping procedures were applied as Mardia’s [27] normalised estimate of multivariate kurtosis was 105.44 (critical ratio 28.16), and values greater than 5.00 are thought to be non-normally distributed [5]. The overall fit of the model was evaluated using the Bollen-Stine corrected *p* value.

CFA was conducted using IBM AMOS 20, [3] employing the Maximum Likelihood Estimation procedure. CFA was conducted to assess the fit of the previously reported five factor model within a clinical eating disordered sample. Several different goodness-of-fit indices were employed, including the significance of *χ*^2^, the normed chi-square, Tucker Lewis index (TLI), Incremental fit index (IFI); the Root Mean Square Error of Approximation (RMSEA), and the Comparative Fit Index (CFI). An RMSEA value of <0.08 indicates a good fitting model [10], with values between 0.08 and 0.10 indicating a mediocre fit [26]. For the remaining fit indices, a value >0.90 is regarded as an acceptable fit of data (e.g., [24, 28, 29]). Factor loadings over 0.40 [17] are considered appropriate.

In order to address the second aim, the mean CET and EDE-Q Global and subscale scores were compared across the clinical and non-clinical samples using a series of Mann Whitney U Tests. To reduce the risk of Type I errors, an alpha level of 0.01 was taken as significant. A binary logistic regression was also employed to assess the discriminative validity of the CET in distinguishing between clinical and non-clinical participants.

Finally, to investigate the third aim, the distribution of CET Global Score was explored and found to be normally distributed on visual inspection of the histograms and according to Kolmogorov Smirnov tests (*D*(716) = 0.03, *p* ≥ 0.05). Receiver operating curve (ROC) analysis was subsequently employed to assess the ability of the CET to distinguish between clinical cases with and without compulsive exercise. ROC is considered to be the gold standard form of analysis in distinguishing cases from non-cases, when using one outcome measure [30]. This form of analysis has previously been used within the eating disorders field to establish the validity of self-report measures and in establishing appropriate cut-off scores [35, 38]. An appropriate cut-off score on the CET was determined through evaluating the sensitivity (the number of correctly identified cases with compulsive exercise) and the specificity (number of correctly excluded patients without compulsive exercise) of the measure at a variety of scores. The Positive Predictive Values (PPV) at the various cut-off scores were also calculated and evaluated. A series of Mann Whitney U tests were conducted to compare CET and EDE-Q scores for those scoring above and below the identified cut off.

## Results

### Characteristics of the sample

The mean scores, standard deviations and group comparisons for each of the measure subscales are presented in Table 1.

There were no significant differences across the clinical and control groups with respect to age. The clinical group reported a significantly lower BMI, and significantly higher EDE-Q Global and subscale scores. The EDE-Q scores are similar to those previously reported in clinical and non-clinical samples [1, 37].

### Confirmatory factor analysis for the clinical sample

Factor loadings and 95 % CI bootstrap criteria for the CET items are given in Table 2. Item 8 (*I do not exercise to be slim*) and Item 12 (*I enjoy exercising*) did not meet the expected factor loadings at 0.14 and 0.24 respectively. All of the factor loadings were significant (*p* < 0.001) apart from Item 8 (*p* = 0.016). The confirmatory factor analysis revealed that the clinical group data marginally fitted the five-factor structure of the CET. The model was found to differ significantly from the observed data (*χ*^2^(242) = 786.50, *p* < 0.001), although the other fit indexes marginally met the criteria: RMSEA = 0.080 (90 % CI = 0.073–0.086), TLI = 0.90, IFI = 0.92, and CFI = 0.92. The Bollen-Stine corrected *p* was significant at *p* < 0.001. The analysis was re-run with Item 8 and Item 12 removed, however this did not improve the fit statistics for the model.

**Table 2:** Factor loadings for items included in the 5 factor model

Items	Standardized factor loadings (SE)	Bias corrected 95 % confidence interval	
		Lower	Upper
Avoidance and rule-driven behaviour			
CET9 If I cannot exercise I feel low or depressed.	0.778 (0.03)*	0.712	0.836
CET 10 I feel extremely guilty if I miss an exercise session.	0.908 (0.01)*	0.875	0.932
CET 11 I usually continue to exercise despite injury or illness, unless I am very ill or too injured.	0.844 (0.02)*	0.802	0.879
CET 15 If I miss an exercise session, I will try and make up for it when I next exercise.	0.830 (0.03)*	0.774	0.872
CET 16 If I cannot exercise I feel agitated and/or irritable.	0.900 (0.02)*	0.860	0.929
CET 20 If I cannot exercise I feel angry and/or frustrated.	0.898 (0.02)*	0.863	0.924
CET 22 I feel like I’ve let myself down if I miss an exercise session.	0.849 (0.02)*	0.798	0.889
CET 23 If I cannot exercise I feel anxious.	0.860 (0.03)*	0.802	0.905
Weight control exercise			
CET 2 I exercise to improve my appearance.	0.721 (0.04)*	0.632	0.793
CET 6 If I feel I have eaten too much, I will do more exercise.	0.765 (0.03)*	0.695	0.821
CET 8 I do not exercise to be slim.	0.143 (0.06)	0.034	0.*256*
CET 13 I exercise to burn calories and lose weight.	0.744 (0.04)*	0.665	0.808
CET 18 If I cannot exercise, I worry that I will gain weight.	0.889 (0.02)*	0.833	0.926
Mood improvement			
CET 1 I feel happier and/or more positive after I exercise.	0.824 (0.03)*	0.766	0.873
CET 4 I feel less anxious after I exercise.	0.775 (0.04)*	0.701	0.840
CET 14 I feel less stressed and/or tense after I exercise.	0.774 (0.04)*	0.691	0.841
CET 17 Exercise improves my mood.	0.828 (0.03)*	0.764	0.878
CET 24 I feel less depressed or low after I exercise	0.644 (0.04)*	0.552	0.725
Lack of exercise enjoyment			
CET 5 I find exercise a chore.	0.699 (0.06)*	0.578	0.801
CET 12 I enjoy exercising.	0.247 (0.07)*	0.113	0.375
CET 21 I do not enjoy exercising.	0.918 (0.05)*	0.819	1.032
Exercise rigidity			
CET 3 I like my days to be organised and structured of which exercise is just one part.	0.648 (0.04)*	0.553	0.727
CET 7 My weekly pattern of exercise is repetitive.	0.860 (0.02)*	0.806	0.902
CET 19 I follow a set routine for my exercise sessions e.g. walk or run the same route, particular exercises, same amount of time, and so on.	0.810 (0.03)*	0.754	0.859

*Note* *p < 0.001

### Comparison of clinical vs control group on CET scores

In order to test the second aim, the mean CET Global and all subscale scores of the clinical sample were compared to those of the non-clinical sample using a series of Mann Whitney U Tests. The results are presented in Table 1. There was no significant difference between the two groups on CET-Mood Improvement scores. However, there were significant differences between the two groups on the remaining four subscales (CET- Avoidance; CET- Weight Control; CET - Lack of exercise enjoyment and CET - Exercise rigidity).

A binary logistic regression analysis was subsequently conducted to assess whether scores on the CET subscales could predict group membership (clinical versus non-clinical). The five CET subscales were entered into the binary logistic regression model. The model was statistically significant, *χ*^2^(5) = 209.78, *p* <0.001, and explained 34 % (Nagelkerke *R*^*2*^) of the variance in and correctly classified 72.5 % of cases. The sensitivity of the model was 71.9 %, specificity was 73.0 %, positive predictive value was 72.7 % and negative predictive value was 72.2 %. Of the five CET subscales, all but Exercise Rigidity significantly contributed to the model. Odds ratios are presented in Table 3.

**Table 3:** Logistic regression odds ratios for eating disorder cases and non-cases

	Eating disorder		
	OR	95 % CI	*P* value
Avoidance and rule-driven behaviour	1.89	(1.55–2.30)	<0.001
Weight control exercise	1.35	(1.13–1.61)	0.001
Mood improvement	0.82	(0.66–1.00)	0.05
Lack of exercise enjoyment	2.16	(1.79–2.61)	<0.001
Exercise rigidity	0.957	(0.80–1.15)	0.64

### Evaluating the criterion validity of the CET and determining an appropriate cut-off

The final aim was to determine an appropriate cut-off score to identify compulsive exercise among those with an eating disorder. ROC analysis was employed to assess the ability of the CET to distinguish between clinical cases with and without driven exercise, using the Global CET score. Participants were separated into those who reported recurrent driven exercise behaviour (>3times per week in the last 28 days, *n* = 185) and those who did not (*n* = 171) using the EDE-Q question: “*Over the past 28 days, how many times have you exercised in a “driven” or “compulsive” way as a means of controlling your weight, shape or amount of fat, or to burn off calories*?” This method has been previously utilized for detecting excessive exercise using the EDE-Q (e.g., [35]). A total of 356 clinical participants were therefore included in the ROC analysis. The analysis indicated that the area under the curve was significant: Area (SE) = 0.81, (0.02) *p* < 0.01; [95 % CI of area: 0.77–0.86]. The sensitivity, specificity and positive predictive values of the CET Global Score are shown in Table 4.

**Table 4:** Sensitivity and specificity of the CET Global Score in distinguishing between clinical participants with and without driven exercise, as measured by the EDEQ

Cut off CET Global Score	Sensitivity	Specificity	Positive predictive value
10.00	0.97	0.35	0.62
11.00	0.95	0.44	0.65
12.00	0.92	0.51	0.67
13.00	0.87	0.57	0.69
14.00	0.83	0.64	0.71
15.00	0.78	0.73	0.75
16.00	0.67	0.78	0.76

The ROC analysis indicated that a cut off score of 10.00 would maximise sensitivity of the CET (0.97). However, specificity at this cut-off score was low (0.35), as was the positive predictive value of the cut-off (0.62). A cut-off that optimises both sensitivity and specificity was deemed more appropriate. A value of 15.00 resulted in acceptable values of both sensitivity (0.78) and specificity (0.73), with a positive predictive value of 0.75. This cut-off was therefore deemed appropriate to aid the identification of compulsive exercise within a clinical population.

A series of Mann–Whitney U tests revealed significant differences between clinical patients scoring above and below the CET proposed cut-off of 15. Participants scoring above the cut-off scored significantly higher on all subscales of the EDE-Q and CET, apart from for Lack of Exercise Enjoyment (CET; see Table 5). No significant differences were observed between the groups for age or BMI. A small proportion of control participants also scored above the proposed cut-off of 15. Table 6 shows that these participants present with significantly increased levels of eating psychopathology in comparison to those scoring below the cut-off.

**Table 5:** Characteristics of the clinical groups screening positively (*n* = 191) and negatively (*n* = 165) when employing the proposed cut-off of 15.00 on the CET

	Negative screen mean (SD)	Positive screen mean (SD)	Mann Whitney U (Z)	Effect size (*r*)
Age	28.90 (10.20)	26.52 (9.03)	2.06	0.11
BMI	22.12 (10.07)	20.34 (5.71)	1.49	0.08
EDE-Q				
Restraint	3.43 (1.80)	4.66 (1.40)	6.22*	0.33
Eating concern	3.53 (1.49)	4.28 (1.20)	4.84*	0.26
Shape concern	4.61 (1.31)	5.23 (0.90)	4.86*	0.26
Weight concern	4.27 (1.78)	4.91 (1.01)	3.99*	0.21
Global score	3.96 (1.29)	4.77 (0.88)	6.52*	0.35
CET				
Avoidance	1.31 (1.09)	4.00 (1.02)	15.00*	0.79
Weight control exercise	2.52 (1.15)	4.30 (0.87)	13.01*	0.69
Mood improvement	2.55 (1.23)	4.08 (0.81)	11.49*	0.61
Lack exercise enjoyment	2.34 (1.26)	1.92 (1.10)	2.86*	0.15
Exercise rigidity	1.67 (1.21)	3.95 (0.90)	14.03*	0.74
Global score	10.39 (3.23)	18.25 (1.92)	16.27*	0.86

*p < 0.001

**Table 6:** Characteristics of the control group screening positively (*n* = 53) and negatively (*n* = 298) when employing the proposed cut-off of 15.00 on the CET

	Negative screen mean (SD)	Positive screen mean (SD)	Mann Whitney U (Z)	Effect size (*r*)
Age	27.11 (8.64)	32.60 (8.38)	4.53*	0.24
BMI	22.11 (4.49)	22.44 (2.68)	1.47	0.08
EDE-Q				
Restraint	0.99 (1.07)	2.23 (1.41)	6.47*	0.35
Eating concern	0.48 (0.72)	1.34 (1.31)	5.76*	0.31
Shape concern	1.60 (1.19)	3.17 (1.71)	6.14*	0.33
Weight concern	1.26 (1.09)	2.67 (1.66)	5.97*	0.32
Global score	1.08 (0.86)	2.36 (1.36)	6.63*	0.35
CET				
Avoidance	1.42 (1.04)	3.56 (0.83)	10.21*	0.55
Weight control exercise	2.38 (1.05)	3.91 (0.84)	8.75*	0.47
Mood improvement	3.11 (1.11)	4.34 (0.66)	7.72*	0.41
Lack exercise enjoyment	1.49 (1.11)	1.10 (0.68)	1.86	0.10
Exercise rigidity	2.16 (1.14)	3.70 (0.74)	8.60*	0.46
Global score	10.57 (2.83)	16.61 (1.23)	11.61*	0.62

*p < 0.001

## Discussion

The aim of this study was to test the psychometric properties of the CET among a group of adult patients with eating disorders. The aims were threefold. First, to determine whether the previously reported factor structure of the CET, derived from a community sample, is appropriate for use with a clinical group. Second, to determine where there is a significant difference between the CET scores of eating disorder patients versus controls, and to assess the discriminative validity of the measure in distinguishing the two groups. Finally, to determine an appropriate cut-off score to identify a clinically-relevant CET score.

The confirmatory factor analysis revealed that the clinical data showed a moderate fit to the previously published five factor model [20, 45], providing further support for the multidimensional nature of compulsive exercise [33]. The clinical group scored significantly higher than the non-clinical group on four of the five CET subscales (and the global score). However, the Mood Improvement subscale scores did not differ between groups. The subscales of Weight Control Exercise, Avoidance and Rule-Driven Behaviour and Lack of Exercise Enjoyment were significant predictors of group membership (clinical versus non-clinical), whilst Mood Improvement did not reach significance. A Receiver Operating Curve analysis revealed that a cut-off score of 15 on the CET resulted in acceptable values of both sensitivity and specificity in distinguishing patients with and without features of compulsive exercise.

### Methodological considerations and limitations

There are several considerations that colleagues should make when determining whether to use the CET. First, assessing compulsive exercise is an important component of treatment programmes. It is also important to be able to understand the maintenance factors for compulsive exercise among non-clinical, sub-clinical and at risk groups (e.g., athletes), since it is only then that clinicians can work with patients to understand and reducing those maintaining factors. The CET is the first measure to assess multiple components of compulsive exercise, based on a model that was developed in the context of the eating disorders. However, it should be noted that, as yet, the CET has not been validated against a clinical interview. Second, it is important to recognise that the mood improvement subscale does not distinguish between clinical and non-clinical participants. Given recent literature, this is not unexpected. Specifically, previous research has shown that the mood improvement subscale is not strongly associated with eating psychopathology among several different groups of participants [20, 39, 45]. Therefore, when determining the extent of compulsive exercise within the context of each patient, results from this subscale should be interpreted with caution. Third, while the confirmatory factor analysis was appropriate, it revealed only a moderate fit with the previously reported five factor model and two of the items (8 & 12) had low factor loadings. It is known to be difficult to replicate factor structures within large samples such as the one reported here [23]. As a result, within its current form, the current findings suggest that the CET can be used with clinical groups, however, since this is the first attempt at validating the factor structure within an adult clinical sample, further research is required to replicate the current findings. Specifically, it is recommended that invariance testing is conducted across control and clinical populations (including across different diagnostic groups) to further validate the proposed model. If, upon replication, the factor loadings for items 8 & 12 remain low then it might be advisable to remove these from the scale. In addition, replication within adolescent patients and with samples including men is required.

When considering other methodological limitations, it is important to note that by using self-report measures with such convenience samples, external validity of the study might be compromised. Finally, further studies would be improved by the inclusion of a more thorough assessment of eating disorder psychopathology among the non-clinical group.

## Conclusions

In conclusion, the identified cut off score of 15 successfully discriminated those with compulsive exercise as a feature of their eating disorder. Given that excessive or driven exercise often pre-dates the onset of an eating disorder [13, 14], it is possible that the CET might constitute an empirical way to identify individuals “at risk” of developing an eating disorder. Indeed, control participants who scored above the cut-off demonstrated significantly elevated levels of eating psychopathology in this study. Further longitudinal investigations should look to explore the predictive ability of the CET. This will be particularly important among those groups known to be “at risk”, such as dancers and athletes. Further research is required to determine whether the reported factor structure is consistent across diagnostic groups. Additional longitudinal studies are required to determine whether the CET is able to distinguish at risk community participants who are at increased risk of developing increased levels of eating psychopathology and/or diagnosable eating disorders over time. The identification of clinical levels of compulsive exercise by the CET could potentially offer a mechanism via which patients can be allocated to targeted treatments, specifically tackling compulsive exercise cognition (e.g., [31, 43, 44]). It will be important for future research to determine whether targeted treatment approaches significantly reduce pathological exercise cognitions and reduce relapse and non-response rates.
